# Numerical model to predict and compare the hypotensive efficacy and safety of minimally invasive glaucoma surgery devices

**DOI:** 10.1371/journal.pone.0239324

**Published:** 2020-09-29

**Authors:** Bachar Kudsieh, Jose Ignacio Fernández-Vigo, Rafael Agujetas, Jose María Montanero, Jose María Ruiz-Moreno, Jose Ángel Fernández-Vigo, Julián García-Feijóo

**Affiliations:** 1 Department of Ophthalmology, Puerta de Hierro University Hospital, Majadahonda, Madrid, Spain; 2 International Center of Advanced Ophthalmology, Madrid, Spain; 3 Department of Ophthalmology, San Carlos Clinical Hospital, Health Research Institute (IdISSC), Universidad Complutense, Oftared, Madrid, Spain; 4 Department of Mechanical, Energy and Materials Engineering and Advanced Scientific Computing Institute (ICCAEx), University of Extremadura, Badajoz, Spain; 5 Department of Medical-Surgical Therapy, Ophthalmology Area, Faculty of Medicine, University of Extremadura, Badajoz, Spain; Faculty of Medicine, Cairo University, EGYPT

## Abstract

**Purpose:**

To predict and compare the hypotensive efficacy of three minimally-invasive glaucoma surgery (MIGS) implants through a numerical model.

**Methods:**

Post-implant hypotensive efficacy was evaluated by using a numerical model and a computational fluid dynamics simulation. Three different devices were compared: the XEN 45 stent (tube diameter, 45 μm), the XEN 63 stent (63 μm) and the PreserFlo microshunt (70 μm). The influence of the filtration bleb pressure (Bp) and tube diameter, length, and position within the anterior chamber (AC) on intraocular pressure (IOP) were evaluated.

**Results:**

Using baseline IOPs of 25, 30 and 50 mmHg, respectively, the corresponding computed post-implant IOPs for each device were as follows: XEN 45: 17 mmHg (29% decrease), 19 mmHg (45%) and 20 mmHg (59%) respectively; XEN 63: 13 mmHg (48%), 13 mmHg (62%), and 13 mmHg (73%); PreserFlo: 12 mmHg (59%), 13 mmHg (73%) and 13 mmHg (73%). At a baseline IOP of 35 mmHg with an increase in the outflow resistance within the Bp from 5 to 17 mmHg, the hypotensive efficacy for each device was reduced as follows: XEN45: 54% to 37%; XEN 63: 74% to 46%; and PreserFlo: 75% to 47%. The length and the position of the tube in the AC had only a minimal (non-significant) effect on IOP (<0.1 mmHg).

**Conclusions:**

This hydrodynamic/numerical model showed that implant diameter and bleb pressure are the two most pertinent determinants of hypotensive efficacy. In distinction, tube length and position in the AC do not significantly influence IOP.

## Introduction

Glaucoma is one of the main causes of irreversible vision loss worldwide [1]. The main risk factor for developing glaucoma is an increased intraocular pressure (IOP), usually caused by an insufficient outflow of the aqueous humor (AH) [2]. Consequently, the main objective of glaucoma treatment is to reduce IOP through pharmacological treatment, laser, traditional filtering surgery, or tube implants [3, 4].

Recently, several new surgery implants, known as Minimally-Invasive Glaucoma Surgery (MIGS) have been developed to improve the efficacy and safety of conventional glaucoma surgery [5]. These implants work by increasing filtration of AH, either through its natural route (iStent implant), or by rerouting the AH flow into either the subconjunctival space (XEN implants and PreserFlo microshunt) or the suprachoroid space (CyPass Micro-Stent implant) [6].

Several studies have assessed the hypotensive efficacy of these MIGS implants, although with relatively short follow-up periods. Data from clinical studies indicate that the hypotensive efficacy varies widely depending on the type of the implant [7–12]. Reference values in order to preoperatively predict the highest likelihood reduction in IOP after implantation of these MIGS devices are not available, but could be very useful to help select the device.

Despite the good safety profile of MIGS, this surgery is associated with risks of several complications, including the loss of corneal endothelial cells, a severe adverse effect that led to the withdrawal from the market of the CyPass implant (Alcon, Dallas TX, USA) [13, 14]. In this case, a direct association was evidenced between the annual loss of endothelial cells and the position of the implant in the anterior chamber (AC). However, the reasons for endothelial corneal loss after aqueous shunt implantation are multifactorial and the exact etiology remains poorly understood [15, 55, 56].

To better understand the pathophysiology of glaucoma, several different numerical models have been developed to study the hydrodynamics of aqueous humour in the AC. These models analyze the parameters that potentially influence AH flow from its production site in the ciliary body to its exit through the trabecular meshwork [16–18] in healthy eyes [19] and after glaucoma surgery [20, 21]. Since they provide a better understanding of the mechanism underlying MIGS implants, these mathematical models could help to optimize glaucoma surgery by predicting the efficacy and safety of this procedure [21, 22].

In this context, the objective of the present study was to predict and compare the efficacy and safety of three different MIGS devices through a numerical model. To do so, we assessed the role of various parameters—baseline IOP, the filtrating bleb pressure, and the implant characteristics [tube length, lumen and position in the AC]—on postoperative IOP.

## Material and methods

### Glaucoma implants and parameters studied

Three different MIGS implants were evaluated: two XEN implants (respectively, with lumens of 45 μm and 63 μm; Allergan Inc. Dublin, Ireland), and the PreserFlo MicroShunt (70 μm lumen; Santen Pharmaceutical Co., Osaka, Japan). Table 1 describes the main characteristics of these devices.

**Table 1:** Characteristics of the glaucoma implants studied in the numerical model.

Implant	Tube lumen (μm)	Total length of the implant (mm)	Tube thickness (μm)
XEN 45	45	6	150
XEN 63	63	6	220
PreserFlo	70	8.5	350

XEN 45: XEN 45 gel stent; XEN 63: XEN 63 gel implant (both from Allergan Inc. Dublin, Ireland); PreserFlo: PreserFlo MicroShunt, (Santen Pharmaceutical Co., Ltd., Osaka, Japan).

The hypotensive efficacy of the implants was calculated using baseline IOP values of 25, 30, and 50 mmHg. The influence of various filtration bleb pressures (*p*_*b*_ = 2, 5, 10, 17, 25, 34 mmHg) was also assessed. We calculated *p*_*b*_ using the formula given below (Formula 1, analytical model section), based on data from clinical studies [7–12].

The following simulations were performed: *p*_*b*_ = 2 mmHg: one-day after implantation; *p*_*b*_ = 5 mmHg, one-week after implantation. Gardiner et al. developed a theoretical model in which a *p*_*b*_ = 17 mmHg was proposed to simulate no scar in the bleb and *p*_*b*_ = 34 mmHg for the presence of scar formation in the bleb [20].

The influence of tube length on hypotensive efficacy was assessed by comparing the standard length tube to a tube that had been shorted by 1 mm. We performed the simulation using three different implant positions in the AC and two different positions in the AC relative to the iris (200 and 600 μm above the anterior iris).

#### Computational simulation

A three-dimensional (3D) simulation based on computational fluid dynamics was performed using the Ansys Fluent software (v6.3.26; Ansys, Inc., Canonsburg PA, USA). The simulations were run in steady regime with laminar flow, using a constant flow rate of 2.0 μl/min of AH [23] as an input condition, with a pressure of 15 mmHg (20 Pa) as an output condition [20]. The fluid was Newtonian and incompressible. The AH was removed from the trabecular meshwork at the same rate as it was produced. In this simulation, the thermal convection caused by the difference between the surface temperatures of the cornea (34º C) and the other parts of the AC (iris and crystalline at 37º C) was considered the main factor for flow in the AC [16]. The buoyancy effects due to temperature gradients were modelled with the Boussinesq approximation [24]. A grid sensitivity analysis was performed before final calculations. For this purpose, three meshes of large, medium and fine sized cells were constructed. For the present study, we used only the intermediate mesh (7.5 x 10^5^) because it had the smallest amount of variations (<1%).

**Fig 1:**
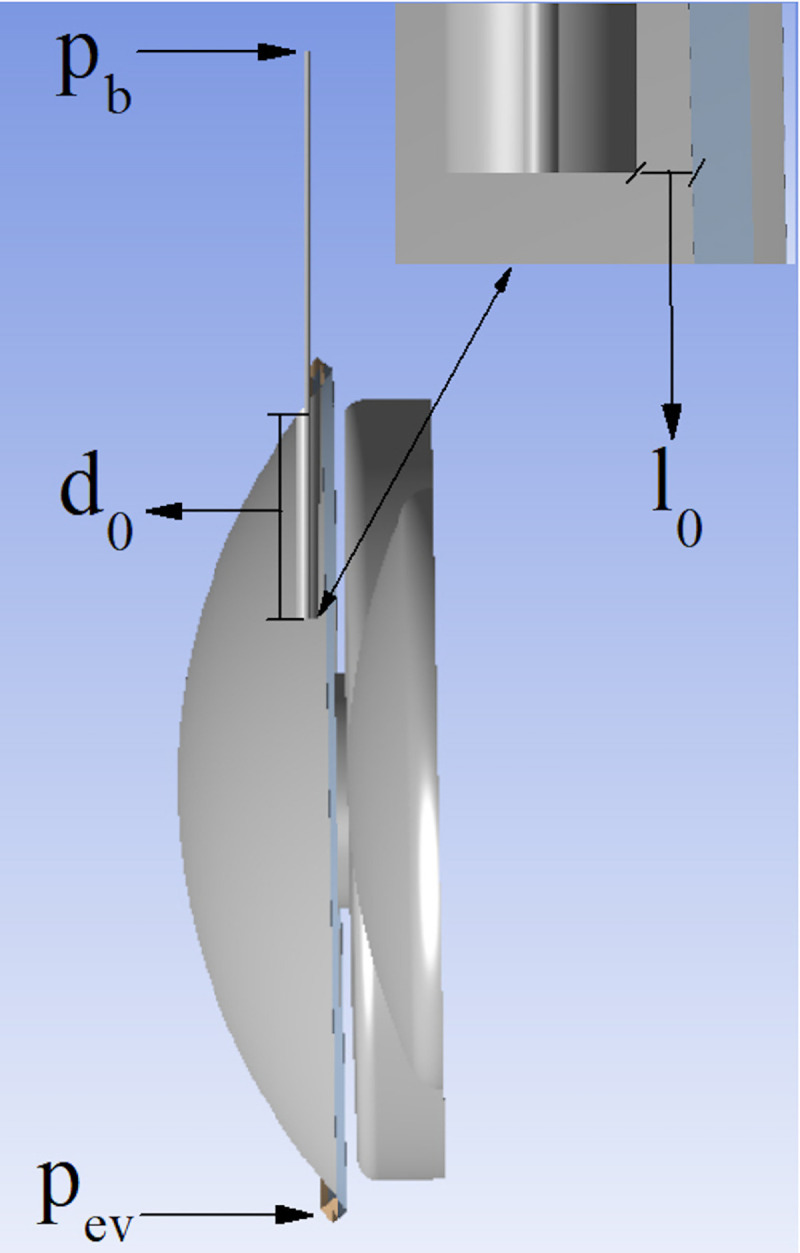
Cross section of the eye showing the parameters related to the implant location in the anterior chamber. L_o_: distance between the implant and the iris, d_o_: length of the implant in the anterior chamber, *p*_*ev*_: pressure in the trabecular meshwork; *p*_*b*_: pressure inside the filtration bleb.

For the elimination of AH, we only considered the trabecular route because this is the main drainage route (accounting for 90% of elimination of AH) [16]; the uveoscleral route was not simulated. Variables included in the simulation were based on standard values for an adult human eye [16, 25–32] (Table 2). The numerical procedure was the same as described by Fernandez-Vigo et al. [33] and Agujetas et al. [34]. However, our simulations incorporated a new element that was not included in those studies: the flow across both the trabecular meshwork and the implanted device. The trabecular meshwork was modelled as a saturated two-phase porous medium [20] whose permeability is calculated as that necessary to obtain the preoperative IOP, *p*_*g*_, for a prescribed pressure *p*_*ev*_ at the exit of the trabecular meshwork. To calculate the trabecular meshwork permeability, we performed the simulation without the implanted device. Once the trabecular meshwork permeability had been calculated, we simulated the flow with the implanted device by imposing pressure *p*_*b*_ in the filtering bleb. The position of the implant is characterized by the distance *l*_*0*_ from the anterior iris and length *d*_0_ of the implant in the AC (Fig 1). This simulation allowed us to determine the quasi-uniform pressure *p*_*c*_ in the anterior chamber following glaucoma surgery, and the flow rate *Q*_*v*_ filtered by the implant. The calculated parameters were *p*_*c*_ and *Q*_*v*_, obtained both analytically and by the 3D simulation software. We also calculated the endothelial wall shear stress (WSS) and the iridian WSS.

**Table 2:** Parameters used for the simulation with the standard values of an adult human eye used in the computational simulation.

Parameter	Value
Diameter of AC, mm	12 [25, 26]
AC depth, mm	3.2 [25, 26]
Diameter of crystalline, mm	9 [27]
Thickness of crystalline, mm	4 [27]
Iris thickness, mm	0.18 [28]
Distance between iris and crystalline, mm	0.93 [25, 28]
Coefficient of linear expansion of AH, b, K^-1^	0.0003 [16]
Density of AH, ρ0, kg/m3	998.2 [29]
Dynamic viscosity of AH; μ, Pa-s	0.001 [29]
Viscosity of aqueous humor at 37 ºC, Pa-s	7x10^-4^ [29]
Thermal conductivity K, W/m-K	0.6 [30]
Acceleration by gravity g, m/s2	981 [31]
Specific temperature Cp, J/kg-K	4182 [32]

AC: anterior chamber; mm: millimeter; AH: Aqueous humor; μ: microns; ρ_0_: AH density; b: Coefficient of linear expansion of AH; K^-1^: 1/Kelvin; Pa: Pascal; ºC: Celsius; W/m-K: Watt/meter-Kelvin; J/kg-K: Joule/Kilogram-Kelvin

#### Analytical model

The present analytical model aims to provide useful information for glaucoma surgeons by modelling the MIGS implants in different scenerios in glaucomatous eyes. This information may be helpful for clinical decision-making and to avoid undesired complications described in previous clinical studies [5–12]. Additionally, as demonstrated by previous studies, these analytical models could predict postoperative IOP, similar to the results found in clinical studies [20, 21].

**Fig 2:**
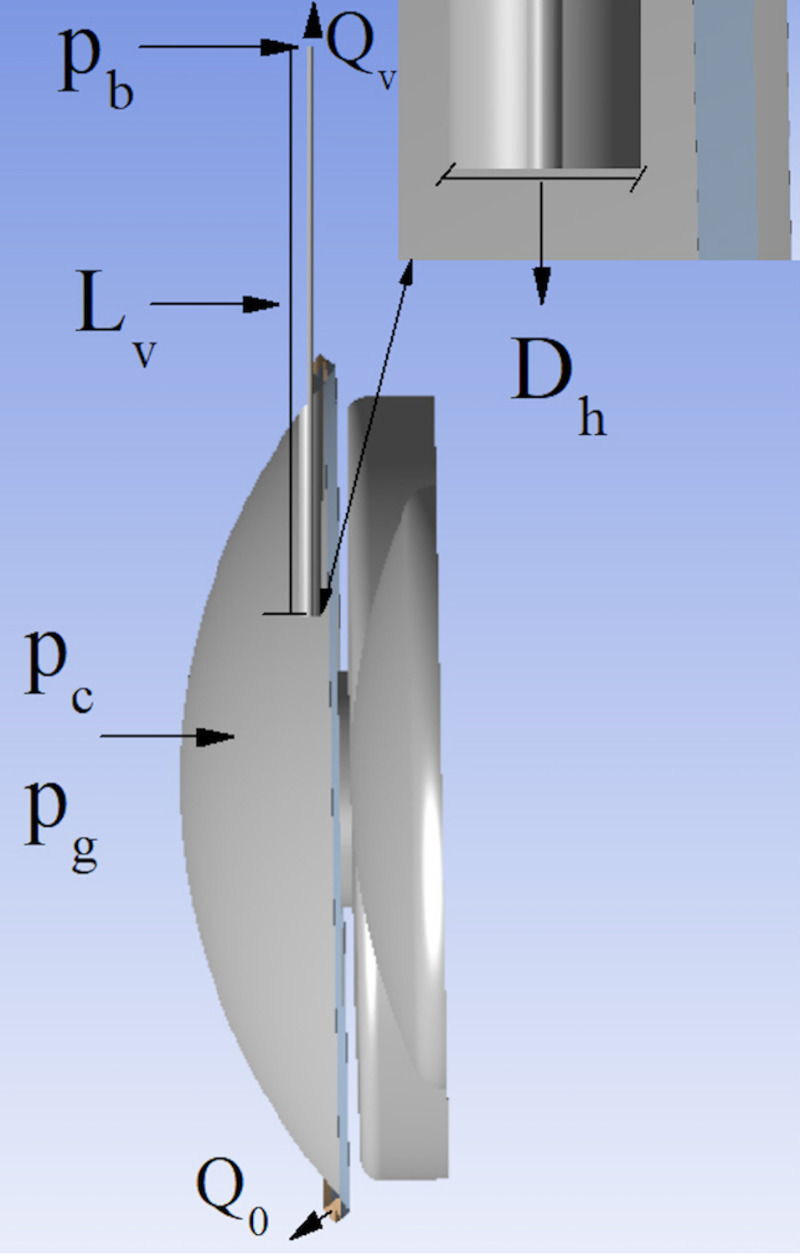
Cross section of the eye showing the parameters calculated by the analytical model. *p*_*c*_: Intraocular pressure (IOP) following glaucoma surgery; *p*_*b*_ IOP following in the filtering bleb; *p*_*g*_: IOP before glaucoma surgery; *Dh*: Implant lumen diameter; *Lv*: Implant total length; *Q*_*v*_: Flow rate evacuated by the implant; *Q*_0_: Flow rate evacuated by the eye before glaucoma surgery.

To validate this model, the parameters were calculated using a computational 3D simulation. The pressure drop across the device obeys the generalized Hagen-Poisuille formula [35] since the Reynolds number characterizing the flow in the implant takes very small values [36], and therefore:

$$p_{c}-p_{b}=\frac{128LL_{v}\mu Q_{v}}{\pi D_{h}^{4}}$$ (1)

where *p*_*c*_ and *p*_*b*_ are the IOP following glaucoma surgery and in the filtering bleb, respectively; L is a dimensionless constant that depends on the shape of the implant cross-section, *Dh* and *Lv* are the implant hydraulic diameter and the total length, respectively; μ is the AH viscosity, and *Q*_*v*_ is the flow rate evacuated by the implant (Fig 2). The trabecular meshwork and Schlemm’s canal jointly provide hydraulic resistance [37]. Because of the smallness of the Reynolds number in this region, that resistance does not depend on the flow rate and can be calculated as the ratio of the pressure drop across this system before surgery to the flow rate *Q*_*0*_ = 1.7 μl/min evacuated by it [38]. This flow rate is calculated as that injected by the ciliary body, *Q*_cb_ = 2 μl/min, minus the flow removed by the uveoscleral pathway, *Q*_up_ = 0.15*Q*_cb_ [39]. The dependency of these two flow rates on the IOP can be ignored [17]. Taking into account these considerations, the IOP following glaucoma surgery, *p*_*c*_, is:

$$p_{c}=\frac{\pi D_{h}^{4}p_{b}(p_{g}-p_{ev})+128LL_{v}p_{g}Q_{0}\mu }{\pi D_{h}^{4}(p_{g}-p_{ev})+128LL_{v}p_{g}Q_{0}\mu }$$ (2)

where *p*_*g*_ and *p*_*ev*_ are the IOP values before surgery and at the exit of the Schlemm’s canal, respectively. The flow rate filtered by the implant is:

$$Q_{v}=\frac{\pi D_{h}^{4}Q_{0}(p_{g}-p_{b})}{\pi D_{h}^{4}(p_{g}-p_{ev})+128LL_{v}Q_{0}\mu }$$ (3)

and coincides with that at which the AH filters from the bleb. The drop of pressure across the implant can be calculated from Eq (2) and the Hagen-Poiseuille formula (1):

$$p_{c}-p_{b}=\frac{128LL_{v}(p_{g}-p_{b})\mu Q_{0}}{\pi D_{h}^{4}(p_{g}-p_{ev})+128LL_{v}Q_{0}\mu }$$ (4)

In this case, the AH moves out the bleb and into the surrounding sub-conjunctival tissue where it is absorbed by the subconjunctival capillaries. Both the sclera and conjunctiva are assumed to be impermeable and thus fluid barriers [40, 41]. Gardiner et al. [20] described in detail the transport and absorption of AH that occurs in the subconjunctival tissue. The flow rate evacuated by the implant, *Q_v_*, under steady conditions verifies the equation:

$$(p_{b}-p_{r})=R_{b}Q_{v}$$ (5)

where *p*_*r*_ is a constant pressure of reference and *R*_*b*_ is a property of the subconjunctival tissue, which provides effective hydraulic resistance. According to the estimates of Gardiner et al., *p*_*r*_ ≃ 0.

#### Influence of thermal convection

**Fig 3:**
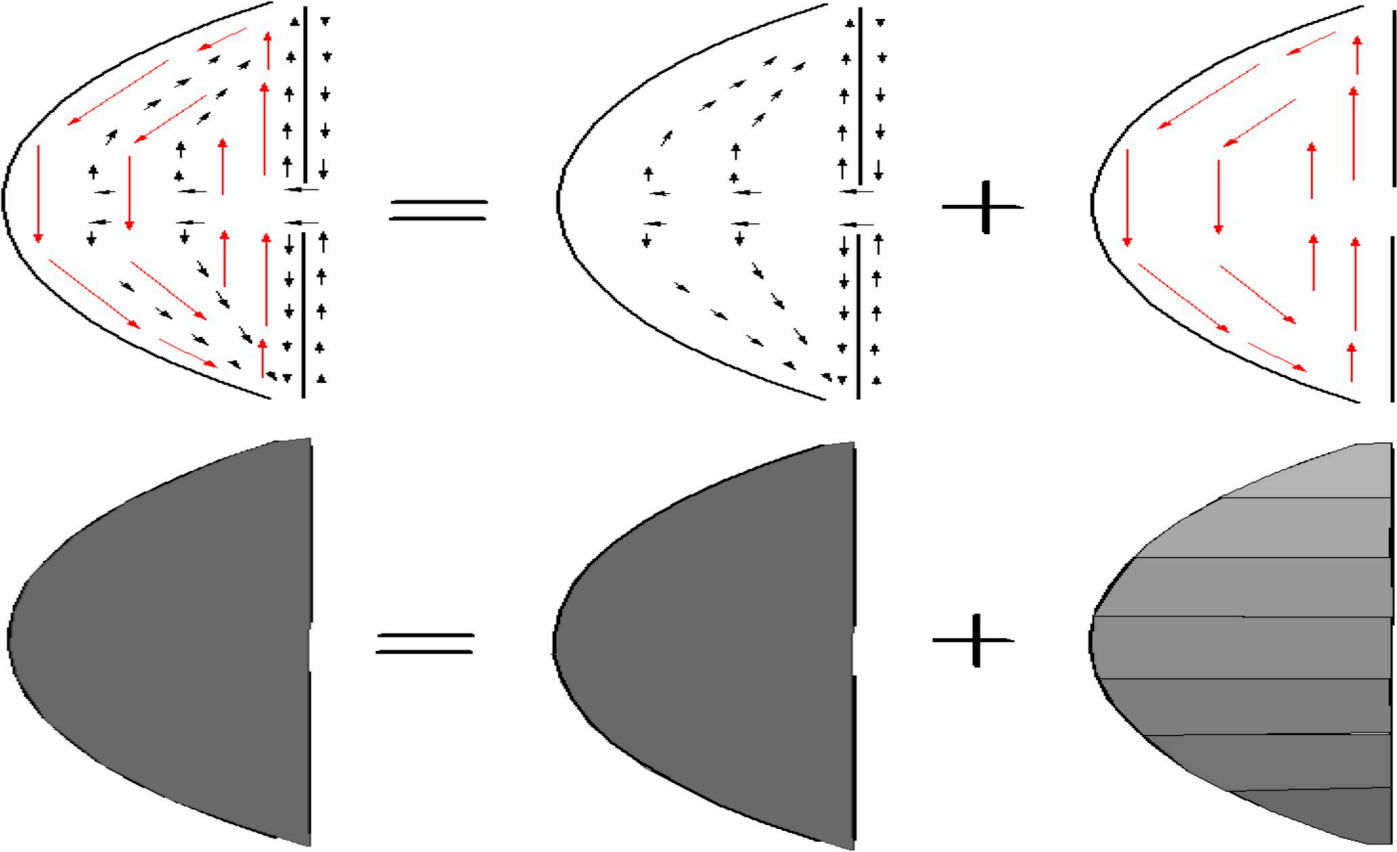
Flow of the aqueous humor in the anterior chamber. Sketch to illustrate the superposition of the two flows in the anterior chamber. The black arrows represent the flow which transports the aqueous humor from the ciliary body to the trabecular meshwork. This flow is driven by a quasi-homogeneous reduced pressure field whose value is determined by the hydraulic resistance imposed by the trabecular meshwork. The red arrows represent the natural convection taking place when the eyelid is open. In this case, the pressure field associated with this flow is inhomogeneous and much smaller than the pressure that is driving the flow across the trabecular meshwork.

In the above mathematical analysis, we have ignored the influence of thermal convection. A simple but accurate way of describing the flow in the AC is as follows (Fig 3): when the eyelid is closed, the AH injected by the ciliary body flows radially from the pupil to the trabecular meshwork. This motion occurs at speeds of 10−4 − 10−3 mm/s [12, 13] at an essentially uniform pressure (the IOP), which ranges from 10–21 mmHg in a healthy eye. By contrast, the IOP usually increases over 21 mmHg and could reach values greater than 30 or 50 mmHg in an eye affected by glaucoma [42]. When the eyelid is open and the body is upright, natural convection occurs in the AC driven by differences between the temperature in the body and the temperature in the posterior cornea. This motion occurs at velocities ranging from 0.1−1 mm/s [15, 16], with an associated dynamic pressure field of approximately 10−4 mmHg. Interestingly, the two flows described above do not essentially interfere with each other due to the disparity between the scales of their velocity and pressure fields. The radial flow does not significantly modify the velocity field of the natural convection, and this convection does not, as assumed by previous studies, alter the IOP [21, 31, 43–45].

## Results

### Efficacy in IOP reduction depending on the baseline IOP

**Fig 4:**
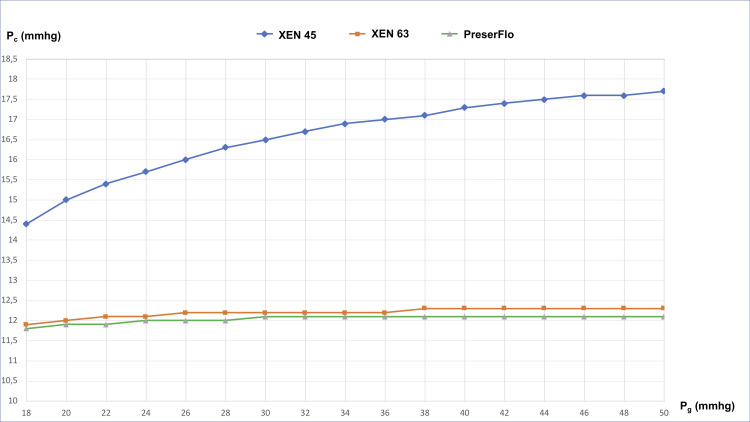
Intraocular pressure after implantation surgery with the three devices. IOP: intraocular pressure; *p*_*c*_: postoperative IOP; *p*_*g*_; preoperative IOP in the anterior chamber; XEN45 (circles), XEN63 (squares), PreserFlo (triangles). In all cases, *p*_*ev*_ = 10.5 mm Hg and *p*_*b*_ = 10 mmHg.

Table 3 and Fig 4 show the post-operative IOP values in the different scenarios. For baseline IOP values of 30 and 50 mmHg (with *p*_*ev*_ = 10.5 mmHg and *p*_*b*_ = 10 mmHg), the IOP (*p*_*c*_) after implantation of the XEN 45 device decreased to 16 mmHg (46% reduction) and 17 mmHg (66% reduction), respectively. For the XEN 63 implant, the IOP decreased to 12 mmHg (60% reduction) and 12 mmHg (76% reduction), respectively. In the PreserFlo implant, the respective values were 12 mmHg (60% reduction) and 12 mmHg (76% reduction).

**Table 3:** Intraocular pressure (IOP) values following glaucoma surgery calculated by the numerical model according to different preoperative baseline IOP values and different bleb pressures.

	*P*_*g*_ = 25 mmHg						*P*_*g*_ = 30 mmHg						*P*_*g*_ = 50 mmHg					
*p*_*b*_	0	5	10	17	20	34	2	5	10	17	20	34	0	5	10	17	20	34
XEN 45–6 mm	12.2	14.8	17.6	20.1	22.4	30.4	11.1	13.1	16.52	21.2	23.2	32.6	13.06	16.7	20.4	23.3	27.8	37.1
Efficacy (%)	51	40	29	19	10	-	63	56	44	29	22	-	73	66	59	53	44	25
XEN 45–5 mm	11.1	13.9	16.6	19.8	22.2	30.8	10.0	12.2	15.7	20.7	22.8	32.8	11.3	15.2	19.1	22.5	26.8	36.6
Efficacy (%)	55	44	33	20	11	—	66	59	47	31	24	-	77	69	61	55	46	26
XEN 63-6mm	5.0	9.0	13.0	18.1	21.0	32.6	5.1	7.8	12.2	18.4	21.2	33.5	4.2	8.7	13.3	18.9	22.5	34.9
Efficacy (%)	80	64	48	18	16	-	83	74	59	38	29	-	91	82	73	62	55	30
XEN 63–5 mm	4.3	8.4	12.5	17.9	20.8	32.8	4.6	7.3	11.9	18.2	20.9	33.6	3.5	8.2	12.8	18.6	22.1	34.7
Efficacy (%)	82	66	50	17	16	-	84	75	60	39	30	-	93	83	74	62	55	30
PreserFlo-8.5mm	4.7	8.7	12.8	18.0	20.9	32.7	4.9	7.6	12.1	18.3	21.0	33.5	3.9	8.5	13.1	18.8	22.3	34.8
Efficacy (%)	81	65	48	18	16	-	83	74	59	39	30	-	92	83	73	62	55	30
PreserFlo-7.5mm	4.2	8.4	12.5	17.9	20.5	32.8	4.6	7.3	11.8	18.2	20.9	33.6	3.5	8.1	12.8	18.6	22.1	34.7
Efficacy (%)	83	66	50	17	18	-	83	74	59	39	30	-	93	83	74	62	55	30

*p*_*g*_: pressure before the surgery; *p*_*b*_: pressure inside the filtration bleb; mm; millimeter; mmHg: millimeter of mercury; %: percentage of pressure reduction over the basal pressure.

The differences between the analytical model and the 3D simulation in terms of efficacy of IOP reduction were <0.02 mmHg in all cases. S1 Table shows the postoperative IOP values in the different scenarios studied.

The flow rate of AH (*Q*_*v*_*)* filtered by the implant calculated by the numerical model for a baseline IOP of *p*_*g*_ 25 mmHg, *p*_*ev*_ = 10.5mmHg and *p*_*b*_ = 15 mmHg was 7.7 μl/min for the XEN 45, 11.45 μl/min for the XEN 63, and 11.56 μl/min for the PreserFlo implant. No significant difference was found between the analytical and simulation flow rate across the three implants in the different positions in the AC (<0.13 μl/min) (S1 Table).

### Effect of the filtration bleb pressure on the hypotensive efficacy of the implants

Table 3 shows the IOP values following implantation of the MIGS devices according to the different *p*_*b*_ values (2, 5, 10, 17, 20, and 34 mmHg) to assess their efficacy at different stages of postoperative scar formation in the bleb.

For a baseline IOP of 30 mmHg, increasing *p*_*b*_ from 5 to 17 mmHg (to simulate the changes that occur in the bleb from the early to the late postoperative period) reduces the hypotensive efficacy from 56% to 29% (8.1 mmHg) in the XEN 45, from 74% to 38% (10.4 mmHg) in the XEN 63, and from 74% to 39% (10.7 mmHg) in the PreserFlo implant.

Interestingly, in cases with a large scar that causes a high *p*_*b*_ (34 mmHg) this may cause inbound pressure towards the AC, thus leading to a postoperative IOP that is higher than the basal IOP.

### Effect of the total length of the implant on hypotensive efficacy

Table 3 shows the IOP values for the shortened implant tubes. A reduction of 1 mm the length of the XEN 45 (16% of the total length) resulted in a decrease in IOP of an additional 1 mmHg compared to the normal tube length, with lower *p*_*b*_ (2, 5,10 mmHg) values, while the reduction was <0.5 mmHg for higher *p*_*b*_ (17, 20, 34 mmHg). In the XEN 63 implant, reducing the length by 1 mm (16% of the total length), decreased the IOP by only 0.5 mmHg. In the PreserFlo implant, reducing the length by 1 mm (11%) led to an IOP decrease of 0.3 mmHg.

### Effect of implant position and length in the AC on *P*_*c*_ and *Q*_*v*_ values

The *p*_*c*_ and flow rate were not affected by the implant position in the AC given by *l*_*0*_ and *d*_0_. The *p*_*c*_ values were very similar in the six scenarios evaluated for each implant. The difference in *p*_*c*_ according to tube position was <0.01 mmHg for all implants, with the following maximum and minimum *p*_*c*_ values, respectively: XEN 45: 19.314 and 19.294 mmHg; XEN 63: 16.665 and 16.659 mmHg; and PreserFlo: 16.657 and 16.547 mmHg (S1 Table).

### Wall shear stress on the endothelium and iris

**Fig 5:**
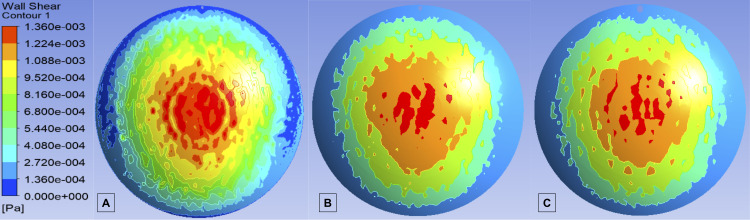
Wall shear stress on the central and peripheral cornea after implantation of the different implants. A: XEN 45; B: XEN 63; C: PreserFlo; Pa: pascal.

Table 4 and Fig 5 show the WSS on the corneal endothelium.

**Table 4:** Values of the mean and maximum wall shear stress following glaucoma surgery for *p*_*g*_ = 25 mmHg, *p*_*ev*_ = 10.5 mmHg and *p*_*b*_ = 15 mmHg.

XEN 45						
l_0_ (μm)	600			200		
*d*_0_ (mm)	1	2	3	1	2	3
*(τw*_*cornea*_*)* (mPa)	0.695	0.696	0.692	0.696	0.689	0.690
*(τw*_*iris*_*)* (mPa)	0.776	0.766	0.762	0.775	0.770	0.767
XEN 63						
l_0_ (μm)	600			200		
*d*_0_ (mm)	1	2	3	1	2	3
*(τw*_*cornea*_*)* (mPa)	0.690	0.690	0.691	0.694	0.691	0.688
*(τw*_*iris*_*)* (mPa)	0.766	0.766	0.762	0.770	0.776	0.766
PreserFlo						
l_0_ (μm)	600			200		
*d*_0_ (mm)	1	2	3	1	2	3
*(τw*_*cornea*_*)* (mPa)	0.692	0.693	0.693	0.695	0.693	0.693
*(τw*_*iris*_*)* (mPa)	0.769	0.760	0.768	0.775	0.766	0.764

*l*_*o*_: distance between the implant and the iris, *d*_*o*_: length of the implant in the anterior chamber, *τw*_cornea_: postoperative wall shear stress (WSS) on the posterior cornea; *τw*_iris_: postoperative WSS following the surgery on the anterior iris; *p*_*g*_: preoperative pressure; *p*_*ev*_: pressure in the trabecular meshwork; *p*_*b*_: pressure inside the filtration bleb; mPa: Milipascal; μm: microns; mmHg: millimeter of mercury.

For *p*_*g*_ = 25 mm Hg, *p*_*ev*_ = 10.5 mmHg and *p*_*b*_ = 15 mmHg, the mean WSS values were 0.690 mPa for the XEN 63, 0.695 mPa for the XEN 45, and 0.692 mPa for the PreserFlo device (all positioned 1 mm in the AC and 600 μm above the iris).

The highest maximum WSS on the posterior cornea was 2.847 mPa with the PreserFlo implant positioned 2 mm in the AC and 600 μm above the iris. Table 4 shows the maximum WSS values on the posterior cornea. The differences between the highest and lowest maximum WSS values for each implant as a function of the position in the AC were 0.189 mPa for the XEN 63, 0.258 mPa for the XEN 45, and 0.669 mPa for Preserflo.

**Fig 6:**
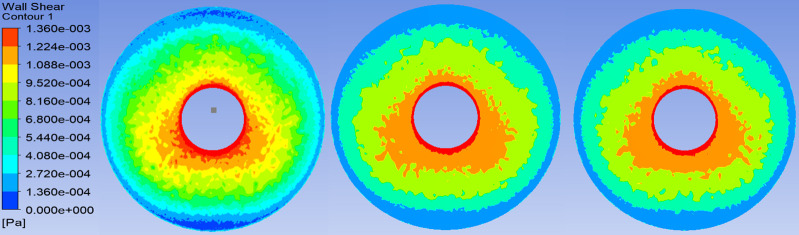
Wall shear stress on anterior iris after implantation of each drainage device. A: XEN 45; B: XEN 63; C: PreserFlo; Pa: pascal.

Fig 6 shows the WSS on the anterior iris. For *p*_*g*_ = 25 mm Hg, *p*_*ev*_ = 10.5mmHg, and *p*_*b*_ = 15mmHg, the mean WSS values were 0.766 mPa for XEN 63, 0.776 mPa for XEN 45, 0.769 mPa for PreserFlo, all positioned 1 mm in the AC and 600 μm above the iris. Table 4 shows the mean and maximum WSS values on the corneal endothelium. The highest WSS values on the posterior cornea was 3.369 mPa, observed in the XEN 45 implant positioned 3 mm in the AC and 600 μm above the iris. The lowest maximum WSS value was 2.945 mPa, in the XEN 63 implant positioned 1 mm in the AC and 600 μm above the iris. The differences between the highest and lowest maximum WSS values for each implant as a function of position in the AC were as follows: 0.288, 0.400, and 0.267 mPa for the XEN 63, XEN 45, and Preserflo, respectively.

## Discussion

Interest in the assessing the efficacy and safety of MIGS implants has increased due to the large number of these devices that have been implanted in recent years [7–12]. In the present study, we applied a hydrodynamic/numerical model to assess the hypotensive efficacy of three common MIGS devices. Our results show that the device diameter is an important factor for hypotensive efficacy, and the Preserflo microshunt is the device with best hypotensive efficacy (due to its larger diameter), followed by the XEN 63 and XEN 45 stents. Similarly, the bleb pressure was found to be a strong determinant of post- implantation IOP.

The clinical studies carried out by Marcos et al. [7] and Ibañez et al. [8] to evaluate the surgical implantation of the XEN 45 both reported similar baseline IOP values (22.2 ± 6.8 mmHg and 22.3 ± 5.4 mmHg, respectively) and a median of 2.5 and 3.4, respectively, hypotensive glaucoma topical medications per patient, which may reduce IOP between 25 to 40% [3]. Thus, in those studies, the real “untreated” baseline IOP may have been as high as 30 mmHg. To facilitate comparison between these studies and ours in term of efficacy, we used the higher baseline IOP estimate. After XEN 45 implantation, those authors reported similar decreases in IOP versus baseline IOP, as follows: 13.2 mmHg (59% reduction) and 12.8 mmHg (57%), respectively on the first postoperative day, and 11.4 (51%) and 11.1 mmHG (49%) at one week post-surgery. Our simulation yielded similar results: in the early postoperative period, IOP decreased by 44% to 56% when the bleb pressure was between 5 and 10 mmHg, respectively.

The decrease in IOP at one month post-implantation in the studies by Marcos et al. and Ibañez et al. was, respectively, 6.5 mmHg (29% reduction) and 6.0 mmHg (26% reduction). At one year after surgery, when the filtration bleb was formed and stable, the respective decreases in those studies were 6.7 mmHg (30% reduction) and 7 mmHg (30% reduction). In most cases, the pressure inside the bleb in the early postoperative period is minimal, only increasing over time due to fibrosis [46–48]. In our simulation, the decrease in IOP ranged from 29% to 22%, while the bleb pressure ranged from 17 to 20 mmHg. It is important to consider that, in routine clinical practice, manoeuvres such as needling of the filtration bleb are usually performed from 6 months to one year after surgery to prevent excessive scar formation, which could lead to more flow resistance in the bleb [49, 50].

In a sample of patients with baseline IOP of 22.5 ± 4.2 mmHg (mean number of glaucoma medications = 2.4) who received a XEN 63 implant, Lenzhofer et al. [10] reported a mean decrease in IOP of 38% (to 14 mmHg) and 34% (to 15 mmHg) at 6 and 12 months after the surgery. Our model yielded a similar decrease in IOP values in the late post-surgery period, ranging from 38% to 29%. Batelle et al. [12] implanted the PreserFlo microshunt in a series of patients, with a decrease in IOP from the baseline IOP (22.1 ± 4.1 mmHg with a mean of 2.9 glaucoma medications) to 8 mmHg (63% reduction) the first week after the surgery and to 10.7 ± 2.8 (55% reduction) one year after the surgery. In our simulation, the calculated decrease in IOP ranged from 74% to 59% and 39% to 30% in the early and late postoperative period, respectively.

Interestingly, Mendrinos et al. measured the length of the Ahmed valve tube in the AC, but did not observe any correlation between tube length and postoperative IOP at 12 months (R = 0.57, P = 0.09) [51]. According to the Poiseuille equation, the lumen and the total tube length of the implant are key factors in the calculation of AH flow [52]. We found that tubes with smaller lumens presented greater output resistance and therefore a lower flow of AH through the tube, which implies a greater postoperative IOP. Thus, in our numerical model, the PreserFlo implant (70 μm) produced the largest decrease in IOP, from 30 mmHg to 12 mmHg (a 60% decrease); by contrast, the XEN 45 μm was the least effective of the devices evaluated, with the model showing a decrease in postoperative IOP from 30 to 16.5 mmHg (45% decrease) Mauro et al. [21] in their theoretical numerical study, observed that the insertion of an Express implant (50 μm) would reduce IOP by 41% (from 25 to 14.7 mmHg).

In our model, a 1 mm decrease in the total tube length would reduce the IOP by less than 1 mmHg, especially when the pressure in the bleb is low. We also found that hypotensive efficacy in the PreserFlo and XEN 63 devices was similar, despite the 2.5 mm difference in total tube length.

The most important factors under normal conditions (production of AH constant) for the calculation of the flow are the lumen and the total length of the tube. Therefore, these two parameters are crucial for implant design to optimize surgical outcomes to ensure adequate efficacy with a minimal risk of hypotony.

We found that the location of the tube in the AC (either more anterior or adjacent to the endothelium, or more posterior and therefore closer to the iris) does not significantly affect the flow of AH through the tube (the difference in *P*_*c*_ according to tube location was <0.01 mmHg for all implants), and therefore, did not affect the postoperative IOP. To our knowledge, in real-life clinical practice no studies have assessed the influence of tube location in the AC in terms of the efficacy of MIGS implants.

In terms of the safety profile of these implants, the magnitude of the WSS on the corneal endothelium and iris surface is an important parameter to assess the risk of cell damage and detachment in these structures. We found that all of these MIGS devices increased the WSS. However, this increase in endothelial and iris WSS is slight, and did not reach the threshold values generally considered necessary to damage to these structures. In our study, the highest WSS values in the central corneal endothelium and iris were 72 and 128 times lower (0.00137 Pa and 0.000775 Pa, respectively) than the values described by Kaji et al. (0.1 Pa) [53] and Gerlach et al. (0.51 and 1.53 Pa) [54] at which endothelial corneal cells become detached. Therefore, the mechanism by which endothelial count decreases after tube implantation in the AC is not related to the flow of the AH but rather is likely to be multifactorial [55, 56].

The differences between the analytical and simulation values for postoperative IOP were insignificant (<0.02 mmHg) in all three devices, a finding that validates our model. Additionally, we found no significant differences (<0.13 μl/min) between the analytical and simulation flow rates in the three implants in six different positions in the AC.

### Study strengths and limitations

This study has several limitations, mainly that the numerical and simulation models use certain variables that are difficult to measure *in vivo*. First, AH production was set as a constant value (2.0 μl/min) even though the flow in the human eye varies during the day, from as high as 2.75 ± 0.63 μl/min [57, 58] to as low as 1.4 ± 0.19 μl/ min during sleep [59]. Second, the bleb pressure used in this study, which is an important determinant of implant efficacy, was simulated based on data from previous clinical studies using our analytical model [7–12], the exact pressure in the filtration bleb during the postoperative period is currently unknown, so future studies with real bleb pressure are needed. Another limitation is that we set AH outflow resistance as a constant in the model, even though this value is not constant in healthy or glaucomatous eyes since many factors can influence outflow, including IOP and the trabecular meshwork, among others [59–61]. Finally, the analytical model does not include the estimation of WSS generated by the AH flow.

## Conclusion

In this study, an improved numerical model and computational fluid dynamics simulation were used to predict and compare the hypotensive efficacy of three MIGS implants. This study shows that the position of the tube in the anterior chamber does not influence IOP. However, the tube diameter and length are both important determinants of postoperative IOP. Of the three implants, the PreserFlo implant produced the largest decrease in IOP and the XEN 45 the smallest. All three MIGS implants were safe in terms of possible corneal endothelium and iris damage.

## Supporting information

S1 Table IOP values after glaucoma surgery and flow rate evacuated by the glaucoma device calculated analytically and in the simulation for *p*_*g*_ = 25 mmHg, *p*_*ev*_ = 10.5 mmHg and *p*_*b*_ = 15 mmHg. (DOCX)
