# Supplementary material for: Numerical model to predict and compare the hypotensive efficacy and safety of minimally invasive glaucoma surgery devices
Source: PLoS One. 2020 Sep 29;15(9):e0239324. doi: 10.1371/journal.pone.0239324 (PMC7523982; doi:10.1371/journal.pone.0239324)
Supplement: S1 Table — (DOCX) [file pone.0239324.s001.docx]

S1 Table. IOP values after glaucoma surgery and flow rate evacuated by the glaucoma device calculated analytically and in the simulation for *p_g_* =25 mmHg, *p_ev_* =10.5 mmHg and *p_b_* =15 mmHg.

| XEN 45 | | | | | | |
| --- | --- | --- | --- | --- | --- | --- |
| l_0_ (µm) | 600 | | | 200 | | |
| *d*_0_ (mm) | 1 | 2 | 3 | 1 | 2 | 3 |
| *p*_c_^(ana)^ (mmHg) | 19.309 | 19.305 | 19.295 | 19.314 | 19.294 | 19.308 |
| *p*_c_^(sim)^ (mmHg) | 19.309 | 19.328 | 19.314 | 19.315 | 19.311 | 19.320 |
| *Q_v_*^(ana)^ (µl/min) | 0.00077 | 0.00077 | 0.00077 | 0.00077 | 0.00077 | 0.00077 |
| *Q_v_*^(sim)^ (µl/min) | 0.00077 | 0.00077 | 0.00076 | 0.00077 | 0.00076 | 0.00077 |
| XEN 63 | | | | | | |
| l_0_ (µm) | 600 | | | 200 | | |
| *d*_0_ (mm) | 1 | 2 | 3 | 1 | 2 | 3 |
| *p*_c_^(ana)^ (mmHg) | 16.664 | 16.662 | 16.663 | 16.664 | 16.665 | 16.659 |
| *p*_c_^(sim)^ (mmHg) | 16.665 | 16.664 | 16.667 | 16.666 | 16.666 | 16.661 |
| *Q_v_*^(ana)^ (µl/min) | 0.00114 | 0.00114 | 0.00114 | 0.00141 | 0.001145 | 0.001141 |
| *Q*_v_^(sim)^ (µl/min) | 0.00114 | 0.00114 | 0.00114 | 0.00114 | 0.001145 | 0.001141 |
| PreserFlo | | | | | | |
| l**_0_** (µm) | 600 | | | 200 | | |
| *d*_0_ (mm) | 1 | 2 | 3 | 1 | 2 | 3 |
| *p*_c_^(ana)^ (mmHg) | 16.555 | 16.547 | 16.550 | 16.566 | 16.552 | 16.567 |
| *p*_c_^(sim)^ (mmHg) | 16.563 | 16.563 | 16.560 | 16.567 | 16.554 | 15.568 |
| *Q*_v_^(ana)^ (µl/min) | 0.001156 | 0.001156 | 0.001153 | 0.001158 | 0.001150 | 0.00116 |
| *Q*_v_^(sim)^ (µl/min) | 0.001150 | 0.001143 | 0.001145 | 0.001158 | 0.001140 | 0.00115 |

*l_o_*: distance between the implant and the iris, *d_o_*: length of the implant in the anterior chamber, *p_c_* ^(ana)^: postoperative IOP calculated by the numerical model; *p_c_* ^(sim)^: postoperative IOP calculated by the simulation; *Q_v_* ^(ana)^: postoperative flow rate calculated by the numerical model; *Q_v_* ^(sim)^: postoperative flow rate calculated by the simulation; *p_g_*: preoperative pressure; *p_ev_*: pressure in the trabecular meshwork; *p_b_*: pressure inside the filtration bleb; µm: microns; mmHg: millimeter of mercury; µl/min: microliter per minute.
